# PULSE: the transformative power of sustained engagement and collaboration to catalyze institutional change

**DOI:** 10.3389/fsoc.2026.1907022

**Published:** 2026-08-28

**Authors:** Michelle D. Withers, Nitya P. Jacob, Taylor Allen, Carlos C. Goller, Gail Hollowell, Matthew R. Marcello, Christopher T. Parker, Michael J. Wolyniak

**Affiliations:** 1 Department of Biological Sciences, Binghamton University, Binghamton, NY, United States; 2 Department of Biology, Oxford College of Emory University, Oxford, GA, United States; 3 Department of Biology, Oberlin College, Oberlin, OH, United States; 4 Department of Biological Sciences, North Carolina State University, Raleigh, NC, United States; 5 Department of Biological and Biomedical Sciences, North Carolina Central University, Durham, NC, United States; 6 Department of Biology, Pace University, New York, NY, United States; 7 Department of Biology, Texas Wesleyan University, Fort Worth, TX, United States; 8 Department of Biology, Hampden-Sydney College, Hampden-Sydney, VA, United States

**Keywords:** change, collaboration, engagement, institutional, Pulse

## Abstract

**Introduction:**

The Partnership for Undergraduate Life Sciences Education (PULSE) works with undergraduate life science departments to enact and sustain reforms in how students engage with STEM in the classroom and laboratory. PULSE works with three signature programs: the Ambassador Program, in which PULSE members work with Departments to develop action plans for short and long-term reform, the Recognition Program, in which Departments self-evaluate their efficacy and are visited by a PULSE team, and the Regional Networks, which foster more local connections between reform-minded institutions. Here, we feature institutions that have engaged with all of PULSE's programs. We seek to better understand how PULSE has served as a catalyst for undergraduate life science education reform across institutions of diverse locations, and missions. Understanding the development of a “culture” for reform, and how the sustainability of reform relies on the infusion of this culture beyond individual change agents to a critical mass of department members, can help develop more impactful professional development interventions.

**Materials and methods:**

Semi-structured interviews were conducted with representatives from three different departments that had engaged with all three of PULSE's programs (25% of all institutions that fit this criteria). Inductive thematic analysis was then used to create a coding scheme and identify similarities and differences between the interviews.

**Results:**

Six key themes emerged between the three departments: 1) motivations for engaging with PULSE; 2) gains from engaging with PULSE; 3) reflecting on change as a process; 4) barriers to change; 5) assistors of change; and 6) perceived impact of multiple interactions with PULSE.

**Discussion:**

Our study preliminarily identifies key commonalities between departments seeking sustainable change to the way they design and implement their curricula despite key differences in institutional size and mission. The work presented here suggests future directions to engage in a more expansive studies to better establish how organizations like PULSE can best catalyze and support systemic reform to undergraduate education.

## 1 Introduction

In 2007, the American Association for the Advancement of Science (AAAS) and the National Science Foundation (NSF) facilitated a conversation with more than 500 faculty, administrators, students, and other stakeholders on how to improve undergraduate biology education. These conversations ultimately led to the release of *Vision* & *Change in Undergraduate Education, A Call to Action* [American Association for the Advancement of Science (AAAS) (2011); referred to as *Vision* & *Change* from this point forward]. The central goal of *Vision* & *Change* was to ensure that undergraduate biology education reflects the way biology is actually practiced by the scientific community and employs research-backed pedagogical strategies to prepare all students—majors and non-majors alike—for the scientific and societal challenges of the 21st century.

*Vision* & *Change* emphasized that undergraduate biology curricula should be built around a set of core concepts and key competencies and taught using proven pedagogical strategies. It also encouraged integrating new tools, such as modeling, simulation, computation, and large databases, into the classroom, while calling for faculty reward systems that properly value teaching and mentoring as a critical contribution to the institutional mission. Taken together, these recommendations represented a cultural shift, urging departments to commit to evidence-based, student-centered learning as a scholarly priority.

The Partnership for Undergraduate Life Sciences Education (PULSE) was established in 2012 to help life sciences departments align with the recommendations of *Vision* & *Change* and facilitate the cultural shift (Musante, 2013; Awong-Taylor et al., 2026). It was launched as a collaborative initiative by program officers from NSF, the Howard Hughes Medical Institute (HHMI), and the National Institute for General Medical Sciences (NIGMS/NIH), and initially it comprised 40 *Vision* & *Change* Leadership Fellows selected across institutional types for demonstrated success as agents of educational reform.

To assist departments with their transformation efforts, PULSE developed three programs designed to support adoption of the *Vision* & *Change* recommendations: 1) the Ambassador Program, 2) the Recognition Program, and 3) Regional Workshops. The Ambassador Program helps STEM departments work together to build inclusive leadership, set collective goals, and design practical plans via intentional self-reflection and collaboration that advance equitable, evidence-based improvements in teaching and learning (Fink et al., 2024). In the Recognition Program, Departments complete a collaborative, rigorous self-assessment process using a series of rubrics designed around the principles of *Vision* & *Change* and the development of strong, inclusive student communities. A team of PULSE members then visits each Department to evaluate the Department's self-assessment, make recommendations, and recognize the Department's achievements through a progression-level model similar to LEED certification (Peteroy-Kelly et al., 2019). Finally, PULSE's five regional networks host a variety of Regional Workshops to foster sustainable communities of practice among institutions, thereby enabling collaboration and sharing progress toward the goals of *Vision* & *Change* (Stavrianeas et al., 2022; Allen et al., 2025).

### 1.1 Intellectual context of PULSE's three programs

PULSE's three programs were conceived simultaneously in an IDEAS Factory sandpit process [called Ideas Lab in the US; for description of the sandpit / ideas lab process, see Collins et al. (2013)]. By tapping into the unique perspectives and insights of diverse participants, the sandpit process aims to elicit transformative ideas and innovative solutions to longstanding challenges. The challenge that PULSE tackled was formulating strategies to shift departmental practices, behaviors, and beliefs in a way supportive of teaching that deepens students' conceptual understanding, develops scientific competencies, sparks wonder and appreciation for science, and meets the nation's need for a scientifically literate citizenry and technically skilled workforce. Motivating this department-level challenge was a growing understanding by the 2000s that curricular innovations, whether designed and proven effective by individual instructors or by groups of institutions, were failing to lead to broader, systemic change in undergraduate STEM education: diffusion and adoption of curricular innovations depended not only on the innovations' demonstrated efficacy, but also on instructors' values, beliefs, and mindset, themselves shaped in part by organizational practices and culture [for review, see for example Graham (2012)]. The shift of attention to faculty members‘ organizational context —the system in which faculty members work—as a strong point of leverage for curricular change is reviewed, for example, by Austin (2011).

The three PULSE programs that emerged from the sandpit process share a focus on departments and seek to develop within them the knowledge capital—skills, relationships, resources, and sense of agency—believed pivotal in catalyzing enduring organizational improvement (Wenger et al., 2011). The ways in which PULSE programs lead to change are being tested empirically and will be reported in the future. The testing is informed by PULSE's Theory of Change (TOC) and by pathway modeling, both of which are explained by Miller et al. (2026). PULSE's TOC is a logic model whose goal is of departments that envision, value, and enact an evidence-based curriculum that aligns with *Vision* & *Change*, and is supported by data-driven program evaluation and departmental policies and procedures. This goal can be achieved through development of eight core capacities, e.g., recognizing and valuing the need for change, knowing, understanding, planning for and enacting processes that will facilitate change, and eight transformational strategies, e.g., leveraging support and collaborations to revise and expand use, evaluation, and communication of equitable, evidence-based educational practices. In short, the PULSE programs collectively advance departments toward the long-term goal of a transformed environment aligned with *Vision* & *Change* and supported by organizational structures, processes, and culture.

The PULSE programs engage departments in the core processes for sustained organizational change through iterative cycles of reflection, visioning, planning, implementation, and assessment. They do so in a complementary fashion with programs emphasizing some overlapping and some distinct aspects of the TOC. For example, the Recognition Program's focus on self-evaluation speaks to a key finding by Graham in her multinational study of change in undergraduate engineering education:

“*Almost without exception, successful and sustainable change starts with a fundamental assessment of the curriculum-wide goals” (Graham, 2012, p. 2)*.

Offering such assessment, the Recognition Program devised, validated, and disseminated the PULSE Rubrics (Brancaccio-Taras et al., 2016). The Program also makes available a site-visit by PULSE Recognition team members, who provide insights and suggestions based on the site-visit and the department's self-study with the Rubrics.

The PULSE Rubrics quantify alignment of programs with inclusive, evidence-based practices pertaining to diverse facets of departmental function: curriculum, assessment (course-level and program-level), faculty practice/support, organizational climate for change, and infrastructure (Brancaccio-Taras et al., 2016). As such, they allow departments to determine strengths and assess gaps in the core capacities and transitional strategies represented by the PULSE TOC and offer a yardstick for measuring change over time (Allen et al., 2025). The Rubrics widen the lens through which departments take stock of themselves and help departments envision a future, improved state.

The Rubrics exist in a full-length version with 77 criteria across 7 broad categories, as well as in an abridged “snapshot” version, with 17 of the most critical items drawn verbatim from the full-length version. The suite of PULSE Rubrics is available at https://pulse-community.org/resources. One criterion pertains to the evaluation of teaching for personnel reviews. For this criterion, a department aligned with inclusive, evidence-based practices would consider teaching as a major component of the overall formal evaluation, and data on an instructor's teaching would be drawn from multiple, diverse sources such as peer evaluations, assessment of students' learning gains, student course evaluations, and a reflective practice striving for continual improvement. This criterion is represented in the PULSE TOC by both a transformational strategy, “use data to diagnose and adjust actions,” and a desired departmental outcome, “data-driven programmatic assessment.”

When used in a departmental self-study, with departmental members collaboratively reaching consensus on scores, Rubrics engender collective responsibility (Fisher et al., 2001) and offer a way to bring to the fore and reflect on the unspoken values and cognitions that shape practices. Such reflection plays a role in shifting an organization from single-loop learning, wherein a problem is fixed, toward double-loop learning, wherein mental models and assumptions underlying the problem are questioned and challenged (Argyris, 1977). The Rubrics represent a tangible form of knowledge capital that complements additional forms of knowledge capital termed human and social (Coleman, 1988; Nahapiet and Ghoshal, 1998; Lesser and Storck, 2001; Wenger et al., 2011). In promoting the emergence and embrace of new mental models, the human and social forms of knowledge capital facilitate collaborative envisioning of an improved future state, a pivotal process in successful transformation efforts (Kotter, 2007). Additionally, beyond the content addressed by the Rubrics, using them in this fashion relates to two core capacities—collaboration and enacting shared leadership—and one transitional strategy—adopting procedures that foster collection of diverse ideas and consensus making—from the PULSE TOC.

Human and social capitals are developed by the Ambassadors Program and the Regional Networks. Social capital grows from relationships: the shared narratives and understandings, the trust and sense of mutual obligation, and the senses of identity, belonging, and companionship experienced during a shared challenge (Coleman, 1988; Nahapiet and Ghoshal, 1998; Lesser and Storck, 2001). Social capital helps a team to be greater than the sum of its constituents. In contrast, human capital is reflected in skills, knowledge, and capabilities, as well as the agency, confidence, and sense of calling to use them (Coleman, 1988; Wenger et al., 2011). As elaborated by Stavrianeas et al. (2022), Fink et al. (2024), and Allen et al. (2025), the human capital developed by the Ambassadors Program and Regional Networks includes skills of creative problem solving (e.g., Treffinger, 1995; Puccio et al., 2011), facilitative leadership (Parnes, 1985; Moore, 2004; Fryer, 2012), systems thinking (e.g., Senge, 2006), and conflict resolution through integrative (“win-win”) problem-solving (Weitzman and Weitzman, 2014). The attention given to particular skills and capabilities differs between the Ambassadors Program and Regional Networks, as well as among the five Networks and their Regional Workshops. The Ambassadors Program and Regional Networks also differ in site of activities. The Ambassadors Program conducts an on-campus workshop for the department hosting an Ambassadors visit, a one-time event. In distinction, with the intention of nurturing an ongoing, sustained community of practice [e.g., Cox (2001), for a focus on higher education, and Wenger and Snyder (2000), for a broader view; for historical review, see Li et al. (2009)], each Regional Network brings together at its Regional Workshops departmental teams from multiple institutions. The myriad ways that they programs support social capital relate to the PULSE TOC core capacity of collaboration and transformational strategy of building connections with other faculty, departments or institutions.

Regardless of skills emphasized and site of activities, the Ambassadors Program and Regional Networks aim to engender the collegial, collaborative interactions through which organizational assumptions are questioned and new mental models concerning effective practices emerge. As new mental models emerge, so do identities (Brown and Duguid, 1991), supportive of deep, structural, and sustained transformation. In this regard, the Rubrics fall short in capturing the full extent of department transformation, e.g., increased motivation, inspiration, empowerment, and agency of departmental members; thus, the Ambassadors Program and Regional Networks monitor their efficacy by using Rubrics as well as interviews, surveys, and products resulting from engagement with PULSE [see Stavrianeas et al. (2022); Allen et al. (2025)].

### 1.2 Research objective of this report

Departments engage with PULSE programs in different ways. Of the 460 departments that have participated in PULSE programs over its history, 3% have engaged with all three programs. The Regional Workshops have served the most Departments since they are often the most convenient and easily-accessible form of engagement for them. As such, the Regional Workshops are often a gateway to other PULSE programs; however, departments that participate in more than one program can do so in any order. Departments with multiple PULSE engagements demonstrate the many ways an institution can work toward alignment with the principles espoused in *Vision* & *Change*. These repeated interactions reinforce that institutional transformation is a continual process that benefits from cumulative engagement paired with emerging local capacity and leadership. To explore the motivations for these engagements and the ways they contribute to departmental transformation efforts, we examined three biology departments that engaged with all three PULSE programs over several years. We report on thematic analysis of in-depth interviews with representatives from these departments.

## 2 Methods

### 2.1 Institutional descriptions/scenarios

To better understand the departmental outcomes associated with extended interactions with PULSE, we conducted interviews with representatives from each of three departments that had participated in all three PULSE programs (Regional Networks, Ambassadors, and Recognition). This sample represents 25% of institutions that have participated in all of PULSE's programming. These institutions are designated as Institution A, B, and C throughout the remainder of this manuscript. We interviewed two individuals from Institution A, three from Institution B, and one from Institution C. In all cases, these interviewees were the liaisons between PULSE and the institution and the individuals who championed reform on their respective campuses. The Departments represented in this analysis are situated within research universities. Additionally, the cohort comprises medium and large; private and public; and institutions located in the northeastern and southeastern United States. We employed a semi-structured interview approach that prioritized a conversational style and allowed for flexibility in following up on responses. This discovery-focused approach allowed the interviewees to tell the story of their transformation journey in their own words. The pre-selected prompts queried: a) department's first encounters with PULSE; b) how the first encounter led to the next interaction; and c) how, if at all, later encounters built on earlier ones. Via thematic analysis (Braun and Clarke, 2006), we inductively coded the interviews and then categorized codes to extract themes. Two authors independently formulated a preliminary coding scheme; then all authors came to consensus on the final scheme. The protocol for qualitative research was approved as exempt by the Longwood University Institutional Review Board via an amendment to the evaluation protocol used by the PULSE Ambassadors Program.

## 3 Results

### 3.1 Reflections on change from departments with extended PULSE interactions

While there were clear differences among these departments, inductive thematic analysis of the interview transcripts resulted in consensus on six major themes: 1) motivations for engaging with PULSE; 2) gains from engaging with PULSE; 3) reflecting on change as a process; 4) barriers to change; 5) assistors of change; and 6) perceived impact of multiple interactions with PULSE. Broadly, these departments all demonstrated a motivation for change, even if that motivation was not shared amongst all faculty; a willingness to take on the challenge of departmental change, even if that change would not come easily; and a perception that interactions with PULSE were beneficial for their change efforts. In the following paragraphs, organized by theme, we will provide a summary and representative examples from the institutions studied.

**Theme 1: motivations for engaging with PULSE**. Regional Workshops were the first encounter for the majority of the interviewed institutions which follows the broader trend of the first engagement with PULSE. When describing that first encounter, the departmental representatives revealed that a common impetus for participating institutions was curriculum reform. “*[W]e had concerns about our current, at that time, current curriculum, and so we were getting together to figure out how you're going to revise it*,” said one interviewee (Institution A, Participant 1). Other motivating factors included having a chair who was familiar with PULSE and perceiving the need for help facilitating the change process. For example, one institution, whose first encounter was with the Regional Workshops, identified inspiration from speakers, excitement over innovative active learning strategies, and exposure to other perspectives as the motivation for soliciting an Ambassador Campus Workshop that helped the department take stock of where they were and get guidance on how to move forward. For the institution whose first encounter was through the Ambassador Campus Workshop, the chair's familiarity with PULSE and desire for aid in a substantial curricular reform of their introductory biology series were instrumental. Despite their reluctance to self-identify as change agents (Fink et al., 2026), local educators who engage in departmental change exemplify the combination of positional/administrative leadership with enabling/adaptive leadership (Uhl-Bien et al., 2007) and change agency (Lunenburg, 2010). When considering all of the interviews collectively, the main motivations for engagement with PULSE included:

- Recognition of the PULSE brand as a proven means of engendering reform.
- Appreciation of inspiration from outside voices to develop plans for change.
- Awareness that curricular or programmatic improvements will be dependent on first changing organizational practices and mindsets.
- Desire for reform momentum to extend beyond a limited number of change agents to a collaborative, coherent, systemic (organizational) effort.

**Theme 2: gains from engaging with PULSE**. All of the departments perceived that their interactions with PULSE were beneficial and cited many different gains from the programs which is not surprising given that they participated in multiple PULSE workshops. Broadly, the respondents identified inspiration, exposure to external perspectives, attitudinal shifts, collaborative skill acquisition, clarification of goals, and mapping of change efforts as positive outcomes from the experiences. While there was some overlap, they related many of these gains to specific programs. For example, the Regional Workshops were recognized as inspiring and useful for exposure to exciting pedagogical innovations, as well as networking and exchanging ideas with colleagues from other institutions. The Ambassador Campus Workshop was seen as key in supporting strategies for improved communication and planning, while the Recognition Program was identified as instrumental for self-assessment in preparation for change planning. Following are specific examples of how these gains played out, categorized by theme. Each thematic header is followed by an illustrative quote relating to that category.

• Ideas/Inspiration/Networking—

“*I also think it [the multiple engagements with PULSE] gave us the external perspective, but also legitimacy.”* (Institution B, Participant 3)

° Successful recruitment of a nationally renowned STEM education reformer whose decision to accept the offer was based in part on the willingness for self-reflection and change signaled by participation in PULSE programming.
° The opportunity to share transformation successes regionally.
° Identification as a reformed teacher and lasting commitment to the use of active learning strategies in place of traditional lecture.
• Communication Skills—

“*I would say it [the Ambassadors program] probably provided a framework for how to have this conversation with all faculty with very different, diverse viewpoints”* (Institution A, Participant 1)

° A framework for productive and inclusive conversations that helped the department navigate diverse views and foster positive attitudes toward change.
° Exposure to the concept of consensus and a process for reaching agreement.
° Guidance for fostering buy-in to change by the department.
• Self-assessment Tools Leading to Change—

“*I got the sense that at least within certain members of the department, there wasn't a good understanding of the functions of the curriculum where we were, there wasn't really an understanding of what kinds of internal assessments were being done, if any. So I think that the first piece [of] doing the Recognition [program], just gathering together all of those data numbers were necessary before even moving forward to figuring out the direction the department wanted to go in.”* (Institution B, Participant 2)

° Self-assessment in preparation for an impending external review.
° Mindset shifts about change that carried beyond the original project.
° Development of a culture of change within the department.

**Theme 3: reflections on change as a process**. An appreciation for change as a continual process rather than a one-and-done situation emerged as a gain for all departments. In the words of one interviewee,

“*I guess I saw it as a sort of gradual process... that initial exposure, the excitement, and then working to try to bring that into the department, and getting buy-in in the department, and then going from there.”* (Institution C, Participant 1).

While viewing departmental change as a gradual, continual process may seem obvious, a shift by STEM education transformers from a deficit mindset approach of “fixing” a discrete problem to a more system's level focus on changing institutional culture has been relatively recent (Luttenberg et al., 2013). In evaluating the long-term impacts of the Ambassador Campus Workshop (Lee et al., 2023; Fink et al., 2026), interviewees deemed changing departmental culture as critical for sustained transformation. Having both of these concepts—viewing change as a process and valuing the need to embed a culture of change in a department—emerge from each conversation without being explicitly solicited was highly encouraging.

This theme emerged slightly differently in the conversations with our three institutions. For two of the institutions, these ideas emerged as they described their change processes including, the need for goal-setting, ongoing meetings, sustained communication, and continued interactions with PULSE. They also reflected on the gradual progression from their first PULSE interaction to their last, revealing a more metacognitive appreciation of the process. One shared their appreciation for sustaining incremental change over time and serving as a role model for other departments on their campus. Another mentioned the importance of systems thinking and the support of local change agents to foster departmental change. The third identified the importance of institutional buy-in and support for the types of culture change needed to enact sustainable change. Similar to participants in the PULSE Recognition Program, they specifically mentioned a change in the department's value of and appreciation for professional development and other reflective teaching processes, e.g., mentoring new faculty, observing one another's classes, sharing assessment reports with colleagues, that signal institutional support for meaningful change. At this intensive research institution, the legitimacy PULSE brought to efforts for improving teaching drove a profound shift in departmental culture. As relayed by one interviewee,

“*[...] I think we're starting to, as a department, value teaching support at all levels, whereas in the past, it has not always been that way. It has been very much a tiered system, where some tiers were not respected by others. And I'm feeling that a lot less in our department. I feel that that was a big culture shift.”* (Institution B, Participant 3).

Outcomes from the Ambassadors Campus Workshops show that both structural and cultural change are needed for sustained change (Lee et al., 2023; Fink et al., 2026) which bodes well for Institution B's change efforts.

When summarizing the interviews with respect to this theme, common trends that emerged included:

- The value of incremental change as essential for sustaining reform.
- The importance of an external organization like PULSE to provide accountability and legitimacy to departments on their journeys of reform, as well as to continue momentum from initial efforts.
- The importance of sustained effort to change mindsets amongst department members and build a culture for lasting reform.

**Theme 4: barriers to change**. While challenges seem ubiquitous for every attempt at change, the stories of barriers faced by our institutions varied greatly. For one institution, no barriers emerged during the conversation, while for another, barriers dominated a large part of the conversation. Two of the three noted disruptions due to the COVID-19 pandemic. In one case the pandemic delayed the time between their second and third engagement with PULSE, which slowed their progress. The other institution experienced significant shifts in activity and continuity with change efforts during the pandemic. Time and workload constraints were barriers that contributed to faculty inability to progress much further than the initial engagement with PULSE. One institution reflected that their use of PULSE was not as efficient as it could have in a more organized environment, while another desired longer-term interactions with PULSE to sustain momentum in challenging times. Two of the three institutions specifically referred to difficulties in maintaining the department-wide culture of reform beyond the initial group of faculty that engaged directly with PULSE. For one of the institutions, significant faculty turnover complicated sustaining the culture of reform. Different levels of faculty buy-in and commitment to the work involved for change were cited as a challenge at another institution. Top-down shifts in priorities at two institutions diverted faculty focus from their original action plans. These shifts include greater emphasis on research and on securing funding for tenure and promotion at one institution, and the addition of new institution-wide programs at another. The following quotes exemplify the challenges of faculty turnover,

“*So the department has changed significantly, and with those changes, the question I have is, how do you take those inspirational things that happened [at the PULSE workshop] and convey them to the next generations of faculty […]”* and shifting departmental foci, “*[...] and again, with a large portion of them not being tenure track […] they have a very different way of thinking about teaching, even in the hiring process*.” (Institution C, Participant 1).

Overall, the major barriers to reform that were cited included:

- Leadership changes in the department and upper administration
- The systemic disruption of the COVID-19 pandemic
- Difficulties in communication among faculty to ensure seamless curricular changes across multiple courses
- Extensive curriculum overhaul complicated by faculty turnover

**Theme 5: assistors of change**. As with barriers, the stories of levers that assisted the change process at our institutions varied widely. In particular, the department that did not mention barriers during the interview cited many assistors, whereas the other departments cited fewer. Levers for change have been shown to be more highly correlated with implementation of new behaviors than barriers (Bathgate et al., 2019) which is an encouraging finding given the seeming ubiquity of barriers. Our institutions found that having a critical mass of faculty willing to embrace change was beneficial to the change process, although lack of critical mass had not been deemed previously a barrier by the PULSE Recognition program (Marley et al., 2026). One interviewee commented,

“[...] *I've been in this kind of work long enough to know we don't want everybody to come anyway. So it was a critical mass, and that mattered to me.”* (Institution B, Participant 1)

Fostering joint effort for change took on many forms. One institution leveraged funding to develop a program that brought faculty together in formally recognized cohorts to support change. Another program intentionally grouped educators from diverse roles, particularly recognizing educational staff as critical to the change effort. In response to this approach, one interviewee shared,

“*So we had, you know, our tenure track faculty, and then we had non tenure track faculty, some instructors. You know, a lot of folks participated. So that was kind of nice, too. It was nice to see common ideas coming out of these different disparate groups.”* (Institution B, Participant 2)

In keeping with one of Laursen's recognized levers (Laursen, 2019), change agents were highlighted as beneficial to supporting reform at the institutions. In some cases, change agents came in the form of people in formal leadership roles, e.g.,

“*[the chair] was really critical in the initial reaching out [...] It's like our chair was looking around and thinking of ways of improving our department and our curriculum.”* (Institution B, Participant 3).

In other cases, new positions were created for change agents. During their sustained engagement with PULSE, one of the institutions hired a nationally renowned change agent to champion their transformation efforts. In our conversations, the change agent cited the institution's involvement with PULSE as a positive factor in their decision to accept the position. For early adopters who followed the lead of a change agent through departmental reorganization, adaptability was highlighted as a key assistor by one institution. For another, external funding was cited as beneficial to both legitimize and financially support the change efforts. Interestingly, some factors deemed barriers by some departments were levers for others. For example, while turnover was responsible for loss of institutional memory for change efforts for one school, for another it acted as a lever by removing faculty who were resistant to change. When considering the entire group, key assistors included:

- Financial compensation for faculty through an internal grant
- Administrative support/buy-in for the need for change
- Obtaining grant funding
- Valuing diverse perspectives from faculty and staff while working on projects

**Theme 6: perceived impact of multiple interactions with PULSE**. In accordance with Bathgate et al. (2019) findings that supports were more related to implementation of new behaviors than were barriers, the list of gains cited from multiple interactions with PULSE was longer for the department that identified no barriers and many assistors. This department also concluded that multiple interactions with PULSE through different programs produced a synergistic effect, while other institutions identified more of a cumulative effect. Each institution reported unique gains, but all agreed that initial contact with PULSE fostered motivation and built momentum, while later interactions provided clarity for specific changes. For example, the institution that started with the Ambassador Campus Workshop identified steps for curriculum reform on their action plan, and their second interaction with PULSE through the Recognition program provided timely feedback on their initial campus change efforts to increase integration and coherence. For another institution, engaging with the Recognition Program after the Regional Workshops set the stage for self-assessment, motivating faculty to gather data, thus enhancing their engagement with the Ambassador Campus Workshop which provided them with further guidance on how to move things forward. The other department that began their change journey at Regional Workshops felt that subsequent interactions with PULSE allowed for continuing incremental change. The interviewee from this department noted

“*that first workshop was inspiring[...]would love to see the lineup of speakers again[...] it was a mind-opening, inspiring period[...]After that, the things that we did within the department, workshop wise, the things that we did with PULSE, workshop wise, built on that for me.”* (Institution C, Participant 1).

This department valued the regional connections for ongoing support. Taken together, key impacts from interacting with PULSE through multiple means included:

- Continuation of momentum gained from the original experience
- Long-term shift in the attitude of the department toward assessment
- Inspiration to learn more ideas from PULSE on best practices for sustainable change
- Grant funding to extend work.

## 4 Discussion

This article represents the first discovery-oriented step for a deeper investigation, through campus case studies, to elucidate the impact of multiple interactions between a department and PULSE. At the level of a whole-case comparison, commonalities emerged regarding the merits of continued engagement with PULSE. All three departments reported experiencing increased benefits from interacting with all the three programs including sustained momentum, cumulative guidance, external legitimacy, and an appreciation for change as a continual process rather than a one-and-done “fix” for a problem. None of the institutions, however, took the same path through the PULSE programs. Institution A started with the Ambassador Campus Workshop, because they needed outside assistance with a formidable curricular overhaul involving core courses, then attended a Regional Workshop and engaged with the Recognition Program last. They noted that the Regional Workshop was beneficial in experiencing innovative curricular approaches and the Recognition Program visit provided timely feedback on the curricular reform that they started with the Ambassador Campus Workshop. Institutions B and C began their journey in the most typical fashion by attending a Regional Workshop. Institution B then engaged with the Recognitions Program followed by the Ambassador Campus Workshop noting the importance of working with the Recognition Program first to provide an assessment-driven approach to their strategic planning and change endeavors developed during the Ambassador Campus Workshop. Institution C highlighted the Regional Workshop as their most beneficial engagement which introduced them to evidence-based authentic research experiences which they later implemented in their curriculum. Based on this positive experience, they hosted an Ambassadors Campus Workshop to take stock of where they were and plan next steps and finished with the Recognition Program that evaluated progress and enhanced implementation of evidence-based educational strategies. They noted the importance of outside inspiration and perspectives in their continued engagement with PULSE.

When examining the way the institutions leveraged their PULSE interactions against their institutional challenges, a “tale of two cities” emerges. Of note, PULSE programs were designed to provide a coherent rationale for transformation while empowering departments to determine the target and scale of their specific change efforts. Therefore, the differences in institutional outcomes observed across the programs reflects not only their unique campus ecosystems but also the varying ways departments leveraged this autonomy. Institutions A and C centered curricular reform and faced by far the most institutional challenges with Institution C citing nearly twice as many barriers as institution A. Both institutions struggled with time and workload pressures and while they had departmental administrative support, they lacked broader institutional buy-in and support which led to external pressures that further diminished their time and support for change efforts. Both institutions felt that their continued engagement with PULSE was additive, rather than synergistic, and cited the pandemic as a significant disruption to the timing and quality of their engagement. Adding insult to injury, both institutions identified very few levers to offset the impact of their barriers.

Alternatively, during the conversation with Institution B, no barriers to their change process arose however multiple assistors were mentioned. This institution engaged with PULSE in response to an external review and used the Recognition Program assessment to provide a “mandate” for their change efforts. They employed a mixed top-down and bottom-up approach that leveraged financial support, both internal and external, to support reformers, institutionalized the support for teaching reform through a new program, hired a nationally renowned change agent to help lead the efforts and included a diverse set of voices and perspectives in the process. This institution described their engagement with PULSE programs as synergistic and noted that they took advantage of the connection with the organization through between-workshop interactions. Despite having participated in all of the PULSE programs, they expressed a strong desire to continue engagement with the organization. Notably, this research intensive institution highlighted a significant change in the culture of valuing teaching that the interviewees admitted they had previously experienced. While making strong conclusions about whether engagement with PULSE programs is more additive or synergistic is contraindicated at this early point in our exploration, it is clear that institutional context including assistors, barriers and stage in the change process, will be a critical contributing factor.

Converting a desire for educational reform in a given department to actionable and sustainable change is a difficult task. Our analysis of these campus transformation stories reveal the development of long-term and evolving engagements between individual departments and PULSE, an outside organization with expertise in evidence-based reform and passion for supporting departments in their reform journeys. For these institutions, PULSE provided external legitimacy and programming that was accessible and collaborative for a critical mass of department members. Through PULSE's programs, champions of reform at each institution were able to engage their colleagues in an evidence-based process of departmental transformation guided by *Vision* & *Change*. The engagement of these departments with multiple PULSE programs in varying order is reflective of the varied needs and goals of each department and the versatility of PULSE's department-centered programming to address varied needs. These long-term, multi-touch relationships demonstrate the continual, complex journey of curricular transformation and how an organization like PULSE can provide the inspiration, tools, and evidence-based framework necessary for success.

Salient across these stories of reform and the six themes extracted from interviews are two features that address the research objective posed at the end of the introduction, namely to examine the motivations for engagement with PULSE and ways repeated engagement supports a program's progress, adaptability, and resilience. First, there is motivation within the department to engage with PULSE and take on the challenge of positive change. As elaborated in the findings pertaining to Theme 1, programs varied in the source of their initial motivation to engage with PULSE: a persuasive, influential person (e.g., chair), curricular concerns held by a few, a wish for outside perspectives, and a recognition of the usefulness of facilitation and conversational frameworks. Yet, as suggested by Theme 5 (Assistors for change), from this seed, the motivation to engage expanded to a critical mass of departmental members as they collaboratively took stock of the department's status, formulated a vision, and planned and pursued paths toward the vision through engagement with PULSE's three programs. Significantly, the motivation stemmed not from a threat to the market position or survival of the program. Such a threat was found to generate the necessary collective urgency for change in the majority of cases studied by (Graham 2012) in her comprehensive examination of undergraduate engineering departments worldwide. For a minority of Graham's cases, the collective urgency was made possible by a culture of collegiality and innovation. We speculate that, like this minority of Graham's cases, programs in our study grew the sense of collective urgency through a culture of innovation and collegiality. In light of Theme 2 (Gains from engaging with PULSE), we further speculate that PULSE's suite of strategies nurtured this culture. Regardless of the departments' starting points and kind of initial engagement, whether through Regions, Recognition, or Ambassadors, the departments saw value in PULSE's offerings and in the kinds of knowledge capital developed through them. Phrased differently, the suite of PULSE offerings was able to “meet departments where they are” and to stoke collaborative, innovative thinking and advancement toward a shared vision.

Indeed, the second feature apparent in these stories of change is a cultural shift within the departments. While most clearly seen in the desire *to sustain* progress toward a shared vision, the shift was also reflected in facets such as interpersonal dynamics (including openness to different or new perspectives), value placed on aligning departmental practices with data-supported ones, and inspiration for innovation (see Themes 2, Gains from engaging with PULSE, and 3, Reflections on changes as a process). These facets speak to social and human capitals pivotal for departmental change [e.g., Allen et al. (2025); in the context of organizational change, see also Coleman (1988), Nahapiet and Ghoshal (1998), Lesser and Storck (2001), and Wenger et al. (2011)]. They also speak to points of positive leverage for change within an interconnected system, such as an academic department (e.g., Meadows, 1997), and lend themselves to “double-loop learning,” wherein mental models and assumptions are examined and challenged (Argyris, 1977). The desire to sustain change efforts went hand-in-hand, in a mutually supportive way, with continued engagement with PULSE, allowing teams to resolve barriers (Theme 4) and tap into assistors (Theme 5). In a similar vein, a previous study of change within programs participating in the Midwest and Great Plains PULSE Network showed that the extent of positive organizational change as detected with scores on the PULSE Rubrics correlated with number of annual Network conferences in which the program participated (Allen et al., 2025). Continued engagement with PULSE can be seen in the light of the Exposure-Persuasion-Identification-Commitment (EPIC) framework to describe buy-in by students and faculty members to curricular innovation (Aragón et al., 2016; Cavanagh et al., 2016). This framework parallels conceptions of the diffusion and adoption of ideas or innovations within a social system (Rogers, 1962). Rogers drew particular attention to the role of culture and norms in the spread and acceptance of new ideas [see pages 57–59 of Rogers (1962)]. From our case studies, as well as other publications on PULSE (e.g., Fink et al., 2026; Marley et al., 2026; Washington et al., 2026), we believe that PULSE supports a program's progress, adaptability, and resilience by helping departments grow their culture of collegiality and research-informed innovation. Moreover, rather than a particular order of engagement with PULSE's programs, sustained interaction with PULSE appears to be key.

Using what we have learned from our current study, we intend to extend our exploration of the impacts of taking part in multiple PULSE programs with a multi-pronged approach. First, we can use Ripple Effect Mapping (REM; Kollock et al., 2012) with the remaining schools who have taken part in all three PULSE programs. REM allows for an extension of the rich qualitative case study approach that emphasizes discovery of the contribution of a program or programs over strict causal attribution due to its Appreciative Inquiry (Cooperrider et al., 2008) approach which is ideal for a small sample size. REM can capture both intended and unintended impacts of engagement with our professional development programs. The themes identified in the current study can inform the focus of interviews and identification of thematic connections between shared stories. The visual mapping element of REM extends beyond simple collection of themes to pathway models that provide insights into the importance of sequencing of actions related to impacts. The benefits of REM is that the “ripples” that arise during mapping sessions can be coded against the six themes from our current project along with the existing PULSE *Vision* & *Change* Rubrics to generate a more robust, comparative metric of departmental transformation across different institutional contexts. It works well with focus-group-style virtual sessions that can positively reengage 10–15 institutions with PULSE without overwhelming the research team.

The second prong of the extended investigation can veer into the quantitative realm using a cohort model and existing rubrics to establish a baseline and post-intervention outcomes. The cohort of study will include institutions who took part in PULSE programs in the same sequence. Since the Regional Workshop is the most common first engagement with PULSE and the Ambassador Program is the second most common program with which departments interact, the first cohort under study will include schools who started with the Regional Workshops and moved to the Ambassador workshop. We can compare outcomes for schools who took part in the Regional Workshop only with those who took part in the Regional Workshop followed by the Ambassador Workshop. We can also reach out to schools with a simple open-ended prompt to determine reasons for schools who stopped after a single intervention as compared to those who chose to re-engage. These extended investigations, informed by the current study, will deepen our understanding of the order and dosage effect of engaging with one or more PULSE programs.

### 4.1 Limitations

This study has several limitations that relate to the qualitative nature of the exploration and the voluntary inclusion of a small sample of campuses drawn from a small total pool. First, although the sample represents a quarter of the total institutions that took part in all three PULSE programs, only three institutions were interviewed for this study. These institutions did, however, represent diverse campus contexts including different regions, research capacities, institutional control, and public engagement missions. Second, participation in the study was voluntary which introduces the possibility for selection bias that may favor institutions with more positive outcomes or stronger connections with PULSE. Third, while the interviewees were key personnel, including chairs and faculty instrumental in the change process, the perspectives shared in the interview were limited to these representatives rather than the whole department. Further, the perspectives shared by the interviewees were subject to both recall bias, given the retrospective nature of the discussions, and social desirability bias to highlight successes over failures or challenges. Our semi-structured interview format sought to offset the potential for social desirability bias by specifically asking about both successes and challenges, noting that this study wished to better understand the process in order to more successfully foster transformation. Additionally, the semi-structured interview format allowed participants to highlight unanticipated themes reducing potential interviewer bias. Institutional change is complex and shaped by many factors, so even without the influence of biases, attempting to attribute such change to particular professional development interventions is difficult, but still can provide insight into better interventions or assessment of change. Finally, the study did not include institutions that participated in fewer programs as a “control” because the goal was to begin a richer, more in-depth qualitative exploration of potentially transferable insights into sustained change rather than seek evidence of statistical generalizability. Limitations notwithstanding, the results of this exploratory multi-case qualitative study suggest common themes that may be explored in more detail to better understand how to sustain evidence-based curricular reform on the backdrop of the complex system of higher education.

### 4.2 Conclusion

So, what can other departments learn from our participating institutions? First, institutional change is a complex process with many contributing factors, so departments will respond somewhat differently to the same intervention based on where they are in the change process combined with their particular local cocktail of levers and barriers. But that doesn't mean we throw our hands into the air and give up. Many studies have contributed to our growing understanding of the common elements and levers for change that have been compiled in key books (Kezar, 2018) and reports (Laursen, 2019). Our departments, like many, come to the change process because of a perceived problem or threat (Rogers, 1962; Graham, 2012). An effective first step could be to identify the desired change and assess the current level of departmental or institution knowledge or buy-in for the change. As such, early efforts by departments at the beginning of the change process may benefit more from opportunities like the Regional Workshops that can provide inspiration along with information, guidance and networking. The guidance from these workshops can aid in identifying pertinent change theories and/or approaches, and acknowledge and foster support for local change agents. Departments further along in the change process may benefit more from interventions like the Recognition Workshops for comprehensive self-assessment and recommendations for next steps or the Ambassador Campus Workshop to adopt distributed leadership and facilitate strategic planning for change. Change projects can take the form of smaller, more concrete “fixes,” termed first order change by Kezar (2018), or more fundamental change that alters the culture of an institution, termed transformational change. While a department might, based on its starting context, i.e., combination of levers, barriers, and point in the change process, achieve transformational change through the engagement with a single professional development program, our departments highlighted the importance of multiple interactions to achieve an appreciation for change as a process that needs to be nurtured by a critical mass of their colleagues along with the continued guidance, legitimacy, and perspectives of external expertise. This is a key element in the quest to achieve comprehensive, evidence-based transformation that does not disappear with turnover in leadership or faculty. PULSE programs are trying to serve this quest by: a) providing a motivational spark, b) helping departments fan the spark into a flame through development of knowledge capital (tangible, human, social), and c) embedding esteem and procedure for stoking the flame in the values, culture, and structure of the department, much the way that the culture of research is integrally embedded in the culture of STEM departments at research institutions.

## Data Availability

The raw data supporting the conclusions of this article will be made available by the authors, without undue reservation.
